# Healthcare resource utilization and cost of pneumococcal disease in children in Germany, 2014–2019: a retrospective cohort study

**DOI:** 10.1186/s41479-023-00105-9

**Published:** 2023-03-25

**Authors:** Tianyan Hu, Bélène Podmore, Rosemarie Barnett, Dominik Beier, Wolfgang Galetzka, Nawab Qizilbash, Dennis Heckl, Timo Boellinger, Jessica Weaver

**Affiliations:** 1 Merck & Co., Inc., Rahway, NJ USA; 2 OXON Epidemiology, London, UK; 3 London School of Hygiene & Tropical Medicine, London, UK; 4 University of Bath, Bath, UK; 5 InGef – Institute for Applied Health Research Berlin GmbH, Berlin, Germany; 6 WIG2 GmbH, Leipzig, Germany

## Abstract

**Background:**

Since the introduction of higher valency pneumococcal conjugate vaccines in 2009, recent estimates on the economic burden of pediatric pneumococcal disease (PD) in Germany have been lacking. This study estimates healthcare resource utilization (HCRU) and medical cost associated with PDs in children < 16 years old in Germany from 2014–2019.

**Methods:**

A nationally representative sample from the Institute for Applied Health Research (InGef) German claims database was used, covering approximately 5% of the total German population. Episodes of pneumococcal pneumonia (PP), all-cause pneumonia (ACP), invasive pneumococcal disease (IPD), and acute otitis media (AOM) in children aged < 16 years were identified using ICD-10-GM codes. HCRU was estimated from annual rates of outpatient visits, outpatient antibiotic prescriptions and inpatient admissions, divided by person-years (PY) at-risk. Average direct medical costs per episode were estimated as the total cost of all HCRU, divided by the total number of episodes. The Mann–Kendall test was used to assess monotonic time trends from 2014–2019.

**Results:**

During 2014–2019, 916,805 children aged < 16 years were followed up for a total of 3,608,716 PY. The average costs per episode for out-versus inpatient care associated with PP and ACP were €67 (95% CI 58–76) versus €2,606 (95% CI 1,338–3,873), and €63 (95% CI 62–63) versus €620 (95% CI 598–641), respectively. For IPD, the average medical cost per episode for out-versus inpatients were €30 (95% CI 19–42) versus €6,051 (95% CI 3,323–8,779), respectively. There were no significant trends in HCRU or costs for IPD or pneumonia over the study period, except for a significant reduction in ACP outpatient visits. A significant decrease in rate of outpatient visits and antibiotic prescribing for recurrent AOM was observed, in addition to an increase in rates of hospital admissions for simple AOM. This was paralleled by a significant increase in inpatient costs per episode for treating AOM overall, and simple AOM, over the study period.

**Conclusions:**

The HCRU and cost per episode of pneumonia and IPD did not vary significantly from 2014–2019, but increased for AOM. The economic burden of pneumonia, IPD, and AOM remains substantial in Germany.

**Supplementary Information:**

The online version contains supplementary material available at 10.1186/s41479-023-00105-9.

## Introduction

Manifestations of pneumococcal disease (PD), caused by *Streptococcus pneumoniae* (*S. pneumoniae),* range from respiratory tract infections, such as non-invasive pneumococcal pneumonia and acute otitis media (AOM), to invasive pneumococcal disease (IPD) including bacteremic pneumonia, bacteremia without focus, and meningitis [1]. Severe disease often results in hospitalization and complications with long term sequelae [2, 3]. An estimated 11–20 million children aged < 5 years are hospitalized per year due to severe cases of pneumonia [4]. In high income countries, it is estimated to account for up to 20% of all pediatric hospital admissions [5]. *S. pneumoniae* is the leading cause of bacterial pneumonia and death in children globally [6–8]. The costs of treating the many manifestations of PD is substantial and varies depending on child vaccination status, clinical manifestation, and other risk factors such as age, and presence of comorbidities [9].

Following introduction of the 7-valent pneumococcal conjugate vaccine (PCV7) in 2000 and 10- and 13-valent PCVs (PCV10, PCV13) in April and December 2009, respectively, a decrease in incidence of PD and associated resource use and cost has been reported throughout Europe and worldwide [10–13]. Although, the cost remains substantial – particularly in children aged < 5 years [9]. In Germany, the PCV has been recommended for all children aged < 2 since June 2006. The initial 3 + 1 vaccination schedule was changed to a 2 + 1 schedule in August 2015 [14]. PCV13 is currently recommended for all children aged < 2 years and children > 2 years at risk of PD due to underlying health conditions [15]. Uptake of PCV has been most recently estimated at 83.4% among children aged 4–7 years in Germany (in 2019, official data published by the Robert Koch-Institut) [16]. Data from the RKI states that approximately 72.5% of infants are fully vaccinated against pneumococci by the age of 24 months, i.e. have received the complete 2 + 1, or, if premature, 3 + 1 schedule. PCV13 is currently used for most infants in Germany [17].

Little published literature exists on the changing economic burden of PD and its various manifestations in the post-PCV era, for children < 16 years old in Germany. Modelling of the cost-effectiveness of PCV13 compared with PCV10 within the German health care system shows reduction of health care resource utilization (HCRU) and cost associated with PD [18]. However, real-world data on the residual economic burden of PD in children are yet to be published following introduction of PCV13.

Currently, new vaccines are under development to further reduce the burden of PD; with a 15-valent PCV recently approved for use in infants, children and adolescents in the US and Europe [19–22]. These novel PCVs contain all serotypes in the currently licensed PCV13 as well as additional serotypes: 22F, 33F for PCV15, and 8, 10A, 11A, 12F, 15B, 22F, and 33F for PCV20. To better understand the current economic burden of PD and the potential value of new vaccines in Germany, it is important to quantify the most recent HCRU and costs following the introduction of PCV13. This study therefore aims to estimate HCRU and costs associated with pneumonia, IPD, and AOM among children < 16 years old in Germany from 2014 to 2019.

## Methods

### Setting

In Germany, health insurance is mandatory; with approximately 88% of the population covered by the public health insurance system, comprising over 100 statutory health insurance providers (SHIs) [23]. The InGef (Institute for Applied Health Research Berlin, formerly Health Risk Institute) claims database includes patient-level, de-identified longitudinal data from around 60 SHIs. Approximately 8 million individuals are included in the database, from across all geographic regions in Germany. A sample dataset of approximately 4 million individuals was used for the present study, covering approximately 5% of the total German population and nationally representative in terms of age and sex [24]. For data protection reasons, individuals can be followed-up within the database over a maximum longitudinal period of six years.

The database includes demographic information, diagnoses and mortality data, hospitalizations, ambulatory services and procedures, and drug prescription and dispensation data. All diagnoses are recorded using the German modification of the 10^th^ revision of the International Classification of Diseases (ICD-10-GM). Claims data for ambulatory services and procedures are reported by the German uniform evaluation standard (EBM,‘Einheitlicher Bewertungsmaßstab’) and procedures conducted in hospital by the German Procedure Classification [OPS, ‘Operationen- und Prozedurenschlüssel ‘]).

### Study design

This retrospective cohort study included children < 16 years of age within the InGef database, between January 1, 2014 and December 31, 2019. Children who were born during the study period were included in the study from their estimated date of birth (the 1^st^ of the respective quarter or the first day of insurance) or the date they started contributing data to the InGef research database. For those children born before 2014, study entry date was assigned as the latter of January 1^st^, 2014 and the date they started contributing data to the InGef research database within the study period.

Six yearly cohorts were established (2014 to 2019). During each study year, each individual was followed-up from the start of the calendar year (January 1^st^) or the date they started contributing data to the InGef research database, until the first of the following censoring criteria: end of observation in the InGef research database (based on: end of insurance with SHI contributing data to the InGef research database, death from any cause), end of study year (December 31^st^) or end of study period (December 31^st^, 2019).

The study population was described by age (< 2, 2–4, 5–15 years) sex (male, female) and underlying medical conditions. Underlying medical conditions linked to higher risk of PD were described according to the 2017/2018 German standing committee on vaccination (STIKO) recommendations for at-risk/high-risk individuals and data availability within the InGef database [15]. Individuals diagnosed with a chronic condition such as diabetes mellitus, chronic pulmonary disease (including asthma), chronic heart disease, or diagnosed with a neurological disorder, were considered at-risk. Individuals diagnosed with cancer, cerebrospinal fluid leak, chronic renal disease, cochlear implant, functional or anatomic asplenia, sickle cell disease/other hemoglobinopathy, congenital or acquired asplenia, splenic dysfunction, splenectomy, HIV infection, immuno-compromising diseases, organ transplant, chronic liver disease and autoimmune disease were considered at high-risk. Underlying medical conditions were assessed in a 12-month retrospective period for each individual from the date of study entry. As no medical history was available prior to 2014, underlying medical conditions were reported only for 2015–2019. The underlying medical conditions were identified by ICD-10-GM codes in the outpatient and inpatient data (all diagnosis fields).

### Exposures

Episodes of PD were identified using ICD-10-GM codes from the outpatient and inpatient data (all diagnosis fields) of the InGef database. Multiple definitions were used for each manifestation of PD (pneumonia, IPD and AOM). Exact code definitions are outlined in the supplementary information.

#### Pneumonia

Two definitions of pneumonia were used, pneumococcal pneumonia (PP) and all-cause (ACP) pneumonia—both of which excluded any IPD. PP was defined as cases where *S. pneumoniae* (pneumococcus) was known to have a causative role as identified from pneumococcal-specific ICD-10-GM codes. ACP was defined as pneumonia cases caused by all unknown and known pathogens (see supplementary information).

#### IPD

IPD included cases of meningitis, bacteremia without focus, and bacteremic pneumonia where pneumococcus was known to have a causative role as identified from pneumococcal-specific ICD-10-GM codes.

#### AOM

Three definitions of AOM were used, AOM overall, simple, and recurrent. AOM included acute suppurative otitis media, suppurative and unspecified otitis media caused by all known and unknown pathogens through ICD-10 GM codes for AOM. Episodes were further classified as simple or recurrent, in alignment with the widely accepted definition of recurrent AOM first coined by Goycoolea in 1991 [25]. An episode of AOM was classified as recurrent if there were three or more episodes within a 6-month period or 4 or more episodes within a 12-month period, with at least one episode in the preceding six months [26]. Episodes not classified as recurrent AOM were classified as simple AOM. To avoid misclassification of a recurrent episode, only children with 6 months of continuous health plan enrolment prior to the first episode and 12 months of continuous health plan enrolment after the first episode were included. The 6-month period prior to the first episode was not required for children < 1 year of age.

For outpatients, only diagnosis by calendar quarter is available in the database therefore at least one prescription of an antibiotic prescription or diagnostic test in the same quarter was required to accompany an outpatient diagnosis of PD. The date of first antibiotic prescription or diagnostic test was assigned as the date of diagnosis. Date of hospital admission was used to assign date of diagnosis within the inpatient data.

Since the analyses were conducted by calendar year, episodes were assigned to each study year. Episodes that crossed calendar years were assigned to the year in which the episode began. For pneumonia and IPD, multiple records were considered as independent episodes if separated by ≥ 90 days [27]. Each pneumonia and IPD episode ended at the last record within the episode plus 90 days. For AOM, multiple records were considered as independent episodes if they were separated by ≥ 14 days [26].

### Outcomes

HCRU and costs were estimated for each PD manifestation. Specifically, HCRU included number of outpatient visits, number of outpatient antibiotic prescriptions, number of inpatient admissions and mean length of stay. The costs associated with each episode included costs associated with each outpatient visit, outpatient pharmacy cost of antibiotic prescriptions during each episode and costs associated with each inpatient hospital admission. Antibiotic prescriptions were captured in the outpatient setting but since indication is not recorded, all antibiotics prescribed during an episode of PD were assumed to be related. Inpatient costs were calculated using the German diagnosis-related groups reimbursement system for hospital admissions, capturing admissions with PD as the main discharge diagnosis [28].

### Statistical analyses

Rate of outpatient visits, prescriptions and rate of hospital admissions were calculated per 1,000 person-years (PY), as total amount of each resource used, divided by total amount of time with PD. 95% confidence intervals (CI) were calculated using the Clopper-Pearson method assuming a Poisson distribution [29]. Annual costs per episode were adjusted for inflation and are presented as 2019 euro values (€) using the consumer inflation index [30]. To assess whether there were any significant monotonic changes in HCRU and costs for each manifestation over the study period, Mann–Kendall linear trend tests were used. All analyses were completed using the statistical software program R, version 3.5.0. If < 5 episodes occurred for any PD manifestation, the data were not shown and HCRU and cost analyses were not conducted, in accordance with InGef’s data protection policies.

## Results

The final study population included 916,805 children aged < 16 years. These individuals contributed a total of 3,608,716 PY at-risk and were followed up for a median of 4.25 (interquartile range 2.0–6.0) years.

At annual cohort entry, the mean age of individuals was 6 years (standard deviation, SD, 5.2) (Table 1). The majority of children were 5–15 years old (52.7%) (Table 1). Nearly 90% of children within each yearly cohort had no history of underlying medical conditions associated with PD (range: 89.2%-90.9% in the 2015 cohort and 2019 cohort, respectively). The most common comorbidity was chronic pulmonary disease, including asthma (range: 5.3% in 2019 to 7.1% in 2015).

**Table 1:** Baseline characteristics of the study population

**All individuals**	**916,805**	
**Year (n)**		
2014	683,807	
2015	682,618	
2016	673,787	
2017	662,069	
2018	654,311	
2019	646,472	
**Age (years) at study entry (mean, SD)**	6	5.22
**Age group (n, %)**		
0–1	306,767	33.46
2–4	126,764	13.83
5–15	483,274	52.71
**Sex (n, %)**		
Male	471,991	51.48
Female	444,814	48.52
Underlying medical conditions for 2015 cohort^*^ **(n, %)**		
No at-risk medical condition	609,198	89.24
Any at-risk medical condition	67,001	9.82
Chronic diseases		
*Diabetes mellitus*	2,089	0.31
*Chronic pulmonary disease (incl. asthma)*	48,728	7.14
*Chronic heart disease*	11,535	1.69
*Neurological disorders*	8,195	1.20
Any high-risk medical condition	9,359	1.37
*Cancer*	931	0.14
*Cerebrospinal fluid leak*	8	0.00
*Chronic renal disease*	848	0.12
*Cochlear implant*	1,078	0.16
*Functional or anatomic asplenia, sickle cell disease/other hemoglobinopathy, congenital or acquired asplenia, splenic dysfunction, splenectomy*	737	0.11
*HIV infection*	27	0.00
*Immuno-compromising diseases*	5,287	0.77
*Organ transplant*	431	0.06
*Chronic liver disease*	408	0.06
*Autoimmune disease*	368	0.05

^*^Underlying medical conditions were assessed in a 12-month look-back period for each individual from the date of study entry. As no medical history was available prior to 2014, results are displayed for the 2015 cohort

### Pneumonia

From 2014 through 2019 the majority of ACP (90%) patients and PP patients (67%) were seen in the outpatient setting (Table 2). The outpatient visit rate was lower for PP compared to ACP, however, the antibiotic prescription rate was similar for both definitions. The rate of hospital admissions was higher for PP compared to ACP; 47% of PP patients were admitted to hospital compared to 21% percent of ACP patients. The mean length of stay per hospital admission was highest for PP at 11 days (SD 20), versus 6 days (SD 10) for ACP.

**Table 2:** HCRU and costs associated with pneumonia and IPD in patients with at least one episode overall during 2014 to 2019

	***PP***	***ACP***	***IPD***
**Total Episodes (n)**	237	67,699	117
***HCRU: Outpatient***			
Number of patients, n (%)	150 (67)	50,601 (90)	24 (29)
Number of outpatient visits (n)	252	87,863	42
Visit rate per 1,000 PY (95% CI)	4,635 (4,080–5,244)	5,801 (5,763–5,840)	1,709 (1,231–2,309)
Number of outpatient antibiotic prescriptions	310	88,796	70
Antibiotic prescription rate per 1,000 PY (95% CI)	5,702 (5,085–6,373)	5,863 (5,824–5,902)	2,848 (2,220–3,598)
***HCRU: Inpatient***			
Number of patients, n (%)	106 (47)	11,854 (21)	72 (88)
Number of hospital admissions (n)	115	14,236	113
Hospital admission rate per 1,000 PY (95% CI)	2,115 (1,746–2,539)	940 (925–956)	4,597 (3,788–5,527)
Mean length of stay per admission, days (SD)	11 (20)	6 (10)	11 (16)
***Costs: Outpatient***			
Visit cost per episode, € (95% CI)	41 (34–48)	42 (41–42)	14 (8–20)
Pharmacy cost per episode, € (95% CI)	26 (22–30)	21 (21–21)	17 (9–24)
Total outpatient cost^a^ per episode, € (95% CI)	67 (58–76)	63 (62–63)	30 (19–42)
Total outpatient cost^a^, €	15,941	4,250,237	3,562
***Costs: Inpatient***			
Hospital cost per episode, € (95% CI*)*	2,606 (1,338–3,873)	620 (598–641)	6,051 (3,323–8,779)
Total hospital cost, *€*	617,532	41,965,738	707,961
**Total inpatient and outpatient cost per episode, € (95% CI)**	2,673 (1,407–3,939)	683 (661–704)	6,081 (3,355–8,808)
**Total inpatient and outpatient cost, €**	633,473	46,215,974	711,523

^a^Outpatient cost including visits and prescriptions

The average out-and inpatient direct medical costs per episode during 2014 to 2019 were greatest for PP, followed by ACP: €2,673 (95% CI 1,407–3,939) and €683 (95% CI 661–704), respectively. Costs per episode were much greater for inpatient versus outpatient care. Outpatient costs per episode were similar for PP and ACP: €67 (95% CI 58–76) and €63 (95% CI 62–63), for PP and ACP, respectively. This was also the case for inpatient cost per episode: €2,606 (95% CI 1,338–3,873) and €398 (95% CI 381–416), respectively. Pharmacy costs per episode were similar for both pneumonia definitions: PP €26 (95% CI 22–30), and ACP €21 (95% CI 21–21). The total inpatient and outpatient cost for PP and ACP, respectively, were €633,473 and €46,215,974, throughout the study period.

The Mann–Kendall tests indicated no significant trends in outpatient visit rate per 1,000 PY and inpatient admission rate per 1,000 PY, except for ACP which showed a significant reduction in outpatient visits over the study period, from 6,283 (95% CI 6,195–6,373) in 2014 to 5,293 (95% CI 5,193–5,394) in 2019, *p* = 0.024 (Table 4). No significant trends were observed for outpatient or inpatient costs per episode for the pneumonia definitions over the study period (Table 5). Similarly, there were no significant trends in rates of antibiotic prescribing over the study period (Table 6).

### IPD

The majority of IPD patients were seen in the inpatient setting (88%) (Table 2). The mean length of stay per hospital admission was 11 days (SD 16).

From 2014 to 2019, the average direct medical cost per IPD episode was €6,081 (95% CI 3,355–8,808). Total outpatient versus inpatient costs per episode were €30 (95% CI 19–42) versus €6,051 (95% CI 95% 3,323–8,779). Pharmacy costs per episode were €17 (95% CI 9–24). The total inpatient and outpatient cost for IPD was €711,523, throughout the study period.

The Mann–Kendall tests indicated no significant trends in HCRU (rate of outpatient visits and hospital admissions), costs or prescribing rates for IPD over the study period (Tables 4, 5, 6).

### AOM

The majority of AOM patients were seen in outpatients, regardless of whether it was simple or recurrent AOM (99%) (Table 3). The outpatient visit rate for AOM overall was 27,811 (95% CI 27,720–27,903) per 1,000 PY; similar outpatient visit rates were observed for simple and recurrent AOM. The antibiotic prescribing rate for AOM overall was 26,895 (CI 26,805–26,985) per 1,000 PY; again, similar rates were observed for simple and recurrent AOM. Hospital admission rates were similar for AOM overall and simple AOM, but higher in recurrent AOM: 673 (CI 659–688), 567 (CI 553–581) and 1,261 (CI 1,212–1,312) per 1,000 PY, respectively. Mean length of stay in hospital for AOM was 4 days, also for simple and recurrent AOM.

**Table 3:** HCRU and costs associated with AOM in patients with at least one episode overall during 2014 to 2019

	***AOM***	***AOM simple***	***AOM recurrent***
**Total Episodes (n)**	327,726	278,715	49,011
***HCRU: Outpatient***			
Number of patients, n (%)	174,244 (99)	173,376 (99)	16,830 (99)
Number of outpatient visits (n)	358,518	303,664	54,854
Visit rate per 1,000 PY (95% CI)	27,811 (27,720–27,903)	27,818 (27,719–27,917)	27,779 (27,547–28,013)
Number of outpatient antibiotic prescriptions	346,703	295,766	50,937
Antibiotic prescription rate per 1,000 PY (95% CI)	26,895 (26,805–26,985)	27,094 (26,997–27,192)	25,796 (25,572–26,021)
***HCRU: Inpatient***			
Number of patients, n (%)	7,410 (4)	5,723 (3)	1,980 (12)
Number of hospital admissions (n)	8,680	6,190	2,490
Hospital admission rate per 1,000 PY (95% CI)	673 (659–688)	567 (553–581)	1,261 (1,212–1,312)
Mean length of stay per admission, days (SD)	4 (9)	4 (10)	4 (8)
***Costs: Outpatient***			
Visit cost per episode, € (95% CI)	30 (30–30)	30 (30–31)	30 (30–30)
Pharmacy cost per episode, € (95% CI)	15 (15–15)	15 (15–15)	15 (15–15)
Total outpatient cost **per episode**^a^, € (95% CI)	46 (46–46)	46 (46–46)	45 (45–46)
Total outpatient cost^a^, €	14,958,011	12,738,847	2,219,164
***Costs: Inpatient***			
Hospital cost per episode, € (95% CI*)*	13 (12–14)	12 (11–12)	20 (18–22)
Total hospital cost, *€*	4,262,479	3,276,294	986,185
**Total inpatient and outpatient cost per episode, € (95% CI)**	59 (58–59)	57 (57–58)	65 (64–67)
**Total inpatient and outpatient cost, €**	19,220,490	16,015,141	3,205,349

^a^Outpatient cost including visits and prescriptions

The average direct medical cost per episode of AOM for outpatients and inpatients during 2014 to 2019 was €59 (CI 58–59); similar results were reported for simple and recurrent AOM. The average outpatient costs per episode were the same for all definitions of AOM: €46 (95% CI 46–46) for AOM overall and simple AOM, and €45 (95% CI 45–46) for recurrent AOM. The average pharmacy outpatient cost was similar for the three definitions, €15 (95% CI 15–15). The total inpatient and outpatient cost for AOM throughout the study period, was €19,220,490.

The Mann–Kendall tests indicated significant decreases in rates of outpatient visits for recurrent AOM and an increase in rates of hospital admissions for simple AOM (Table 4). Similarly, trends indicated a significant increase in inpatient costs per episode for treating AOM overall, and simple AOM but no monotonic trends for recurrent AOM costs over the study period (Table 5). The inpatient cost per episode for AOM and simple AOM increased significantly from €11 (95% CI 10–13) to €18 (95% CI 16–20) (*p* = 0.027), and from €10 (95% CI 8–11) to €17 (95% CI 15–19) (*p* = 0.051), respectively. No significant trends were found for outpatient costs.

**Table 4:** Rate of outpatient visits and inpatient admissions associated with pneumonia, IPD and AOM from 2014 to 2019

	**2014**		**2015**		**2016**		**2017**		**2018**		**2019**		**Test for trend** **(*p*-value)**
	Rate per 1,000 PY	95% CI	Rate per 1,000 PY	95% CI	Rate per 1,000 PY	95% CI	Rate per 1,000 PY	95% CI	Rate per 1,000 PY	95% CI	Rate per 1,000 PY	95% CI	
**Outpatient visit rate**													
PP	6,512	5,614–7,512	6,142	4,228–8,626	7,457	4,555–11,516	5,607	3,835–7,915	8,310	5,909–11,360	5,520	3,537–8,213	0.707
ACP	6,283	6,195–6,373	5,837	5,749–5,927	6,072	5,981–6,165	5,707	5,613–5,803	5,313	5,219–5,407	5,293	5,193–5,394	0.024
IPD	NA	-	3,433	1,828–5,871	NA	-	2,300	1,225–3,933	NA	-	NA	-	-
AOM	27,967	27,761–28,173	27,835	2,7625–28,046	28,127	27,911–28,344	27,736	27,510–27,963	27,572	27,341–27,804	27,479	27,241–27,718	0.060
AOM simple	27,935	27,709–28,162	27,840	27,611–28,071	28,159	27,924–28,396	27,746	27,501–27,993	27,575	27,327–27,826	27,509	27,255–27,766	0.060
AOM recurrent	28,118	27,622–28,620	27,806	27,287–28,332	27,952	27,409–28,503	27,676	27,094–28,267	27,549	26,932–28,177	27,270	26,608–27,944	0.024
**Inpatient admission rate**													
PP	4,510	3,723–5,414	4,715	2,955–7,139	3,789	1,733–7,193	5,111	2,721–8,739	4,884	3,218–7,105	4,767	2,951–7,287	0.452
ACP	918	885–953	873	839–908	901	866–937	941	903–980	996	955–1,037	1,056	1,012–1,101	0.060
IPD	4,300	2,458–6,984	4,226	2,415–6,862	4,226	2,250–7,226	4,423	2,862–6,529	4,730	2,803–7,475	5,466	3,602–7,953	0.085
AOM	655	624–687	668	636–702	644	612–678	665	630–701	684	648–721	739	701–779	0.133
AOM simple	537	506–569	553	521–586	544	512–578	553	518–588	588	552–625	646	607–686	0.035
AOM recurrent	1,224	1,122–1,333	1,263	1,154–1,379	1,186	1,076–1,304	1,303	1,179–1,436	1,282	1,151–1,423	1,383	1,237–1,541	0.133

**Table 5:** Total average cost per episode for inpatient and outpatient care associated with pneumonia, IPD and AOM from 2014 to 2019 (€)

	**2014**		**2015**		**2016**		**2017**		**2018**		**2019**		**Test for trend** **(*p*-value)**
	Cost (€)	95% CI	Cost (€)	95% CI	Cost (€)	95% CI	Cost (€)	95% CI	Cost (€)	95% CI	Cost (€)	95% CI	
**Outpatient**													
PP	81	61–102	66	39–94	89	62–116	101	75–127	71	58–85	121	73–169	0.260
ACP	66	65–67	66	65–67	66	65–66	67	66–68	63	62–64	63	61–64	0.411
IPD	NA	-	53	4–101	NA	-	33	12–55	NA	0–0	NA		-
AOM	47	47–47	47	47–47	47	47–48	49	47–49	48	48–48	47	47–48	0.652
AOM simple	47	46–47	47	47–47	47	47–48	49	47–49	48	48–48	47	47–48	0.652
AOM recurrent	47	46–48	46	46–47	47	46–48	47	46–49	47	46–48	46	45–48	0.840
**Inpatient**													
PP	7,398	-1359–16,156	2,761	1366–4155	5,037	720–9352	3,157	1674–4639	11,777	-1300–24,853	5,327	2516–8138	0.707
ACP	564	516–612	557	513–600	623	567–679	624	581–666	755	693–819	787	714–861	0.840
IPD	3,673	2134–5213	4,690	-423–9803	8,750	-4065–21,566	4,206	1691–6721	4,625	1730–7520	12,703	2128–23,278	0.260
AOM	11	10–13	13	12–14	13	10–14	13	12–15	14	12–15	18	16–20	0.027
AOM simple	10	8–11	12	11–13	10	9–12	12	10–13	13	11–14	17	15–19	0.051
AOM recurrent	18	15–22	17	14–21	23	18–27	23	18–27	21	16–25	28	21–35	0.181

For AOM overall, simple AOM and recurrent AOM, the rates of antibiotic prescriptions (per 1,000 PY) went from 27,097 (95% CI 26,894–27,300) to 26,536 (95% CI 26,302–26,771), from 27,247 (CI 27,024–27,472) to 26,761 (CI 26,510–27,014), and from 26,372 (95% CI 25,892–26,859) to 24,987 (95% CI 24,353–25,632), respectively, from 2014 to 2019 (Table 6). Only in patients with recurrent AOM was this reduction significant over the study period (*p* = 0.008).

**Table 6:** Antibiotic prescription rate in outpatient care associated with pneumonia, IPD and AOM from 2014 to 2019

	**2014**	**2015**	**2016**	**2017**	**2018**	**2019**	**Trend test** **(*p*-value)**
**PP**							
N patients	29	18	27	28	23	32	
Rate per 1,000 PY	5,180	6,525	6,181	5,968	5,598	5,549	0.452
95% CI	3,869–6,793	4,491–9,163	4,602–8,127	4,565–7,666	4,141–7,401	4,334–7,000	
**ACP**							
N patients	11,977	10,712	10,932	9,256	8,328	7,466	
Rate per 1,000 PY	6,363	5,893	6,125	5,750	5,367	5,380	0.060
95% CI	6,274–6,454	5,804–5,983	6,033–6,218	5,655–5,846	5,273–5,462	5,280–5,482	
**IPD**							
N patients	NA	6	NA	8	NA	NA	
Rate per 1,000 PY	-	4,490	-	3,185	-	-	1
95% CI		2,616–7,189		1,887–5,033			
**AOM**							
N patients	45,636	44,148	43,050	38,830	37,348	35,269	
Rate per 1,000 PY	27,097	26,909	27,230	26,774	26,636	26,536	0.060
95% CI	26,894–27,300	26,703–27,117	27,018–27,444	26,552–26,998	26,410–26,865	26,302–26,771	
**AOM simple**							
N patients	43,852	42,574	41,480	37,459	36,145	34,223	
Rate per 1,000 PY	27,247	27,092	27,465	26,997	26,842	26,761	0.060
95% CI	27,024–27,472	26,866–27,320	27,233–27,699	26,755–27,241	26,597–27,089	26,510–27,014	
**AOM recurrent**							
N patients	4,983	4,562	4,273	3,656	3,268	2,546	
Rate per 1,000 PY	26,372	25,966	25,962	25,511	25,357	24,987	0.009
95% CI	25,892–26,859	25,465–26,474	25,439–26,493	24,953–26,079	24,765–25,960	24,353–25,632	

## Discussion

This study provides insights into the HCRU and costs of PD in Germany between 2014 and 2019 for children aged < 16 years. Among children in Germany, pneumonia was predominantly treated in the outpatient setting, while IPD was predominantly treated in the inpatient setting. The rate of hospital admissions and inpatient cost per episode were greatest for IPD, followed by PP and ACP. Outpatient visit rates were highest for ACP, and outpatient costs per episode were greatest for ACP and PP. There were no significant monotonic trends in HCRU or costs for IPD or pneumonia over the study period, except for a significant reduction in ACP outpatient visit rates. In the case of simple and recurrent AOM, treatment was almost always in the outpatient setting. Hospital admission rates were two-fold higher in patients with recurrent AOM compared to patients with simple AOM. Over the study period a significant decrease in rate of outpatient visits and antibiotic prescribing for recurrent AOM was observed, in addition to an increase in rates for hospital admissions for simple AOM. This was paralleled by a significant increase in inpatient costs per episode for treating AOM overall, and simple AOM, over the study period. Despite the introduction of PCV13, the economic burden of PD continues to be substantial in Germany.

Prior modelling of the cost-effectiveness of PCV13 compared with PCV10 within the German health care system predicted reduced HCRU and cost associated with invasive and non-invasive disease following PCV13 introduction, but an increase in HCRU and cost associated with AOM [18]. This is broadly in alignment with results reported in the present study. Although we saw no significant change in the annual cost per episode for treating pneumonia and IPD in the inpatient or outpatient setting, there was a significant decrease in outpatient visits for ACP. We also observed an increase in outpatient visit rates for recurrent AOM, in addition to an increase in inpatient admissions for simple AOM and increase in inpatient costs associated with AOM (overall and simple). This could be due to an increasing incidence of AOM complications. Previous studies have reported an increasing burden of AOM-related complications in the post-PCV era [26, 31–36]—perhaps due to shifts in AOM etiology or pneumococcal serotype distribution as a result of PCV introduction.

Previous studies reporting HCRU and cost of PD in children in Germany following PCV introduction are limited. Earlier studies documenting cost of childhood PD in Germany before the PCV-era are also lacking. One study explored the economic impact of lower respiratory tract infections, including pneumonia, in infants aged 0–36 months from 1999–2001 [37]. Direct medical costs per case of pneumonia were estimated at €85 and €2,306 for office-based pneumonia cases (treated by office-based pediatricians) versus hospitalized, respectively. Results from the present study would perhaps suggest a reduction in costs in comparison to our study period: outpatient cost per episode of ACP estimated at €40 (95% CI 39–41) and €44 (95% CI 44–45) for children aged < 1 and 2–4, respectively (results not presented), versus €1,225 (95% CI 1,1600–1,289) and €513 (95% CI 484–542) for hospital costs per episode of ACP. However, direct comparisons between studies can be problematic, due to different cost definitions, age groupings or even coding definitions.

Comparisons with literature from other countries is similarly challenging. Costs per episode for treating PD vary significantly from country to country depending on healthcare system. For example, total costs (including indirect societal costs) per AOM episode have been reported to range from €332.00 in the Netherlands to €752.49 in the UK [38]. Beyond Germany, Shiri et al. recently reviewed the annual HCRU spend on PD including IPD, pneumonia and AOM from 2010 to 2018 in high-income countries and showed an increase in cost and resources used (including both out- and inpatient care) [9]. In addition, a study conducted in the Veneto region of Italy from 2010–2017 in children < 15 years old [39], showed that IPD burden did not change significantly following PCV13 introduction, while disease burden declined for outpatient PP. Average cost per episode was reported for pneumonia and IPD as €345 and €4,206, respectively. Similarly, a study within the Liguria region of Italy reported an increase in the number and cost of emergency department visits and hospitalizations associated with IPD, ACP and AOM in children aged < 15 years between 2012–2018, while costs associated with PP decreased [40].

The data from this study demonstrate that the economic burden of PD in children in Germany remains substantial following introduction of PCV13. The cost-effectiveness of PCVs in children is evident from the literature [41–44]. However, in recent years there has been an increase in cases of PD associated with serotypes not included in PCV13. A study in 2017 found that non-PCV13 serotypes accounted for over 70% of IPD in children in Europe [45]. Indeed, a recent study in the UK reported that although overall incidence of IPD was lower in 2016/2017 (9·87 cases per 100 000) than pre-PCV7 (14·79 per 100 000; 37% lower) and pre-PCV13 (10·13 per 100 000; 7% lower), incidence of IPD due to non-PCV13 serotypes had doubled (from 3·85 to 7·97 per 100 000), and accelerated since 2013/14—especially serotypes 8, 12F, and 9 N [46]. Similar trends have been reported for other PD outcomes [47–51]. The continued clinical as well as economic burden of PD after the introduction of PCV13 demonstrated in this study, highlights the need for novel vaccines targeting additional serotypes to reduce the incidence, HCRU and costs associated with PD in children.

There were several limitations to this study. Firstly, although the InGef database is representative of the German population in terms of age, sex, morbidity, mortality and drug usage [24], it is unclear whether it is representative in terms of factors such as ethnicity and socioeconomic status. As a statutory (public) health insurance claims database, it is possible that the population contributing data to InGef may differ from the general German population regarding socioeconomic status, as wealthier individuals may opt for private insurance [52]. Secondly, there is also a potential risk of misclassification bias due to coding inaccuracies, as medical conditions were identified based on existing records. However a prior Danish study found good agreement between PD such as AOM recorded via ICD-10 codes and parent-reported prior diagnosis of PD [53]. Furthermore, a prior study in the US found 94% agreement between ICD-10 coded AOM and manual review of the medical record [54]. ICD-10 or ICD-9 codes have been used to identify PD diagnosis in many prior studies assessing incidence of various PD outcomes, including recent studies in Germany and the US [55, 56]. Despite this, pneumococcal HCRU and cost burden for PP and IPD are potentially underestimates, due to likely under-identification of PP and IPD episodes. Recent studies in children have suggested that using administrative databases to assess organism-specific prevalence in pneumonia and other conditions may underestimate true organism-specific burden – potentially leading to a corresponding underestimation of PP and IPD economic burden in the present study [37, 57, 58]. Pathogen-specific pneumonia codes lack adequate sensitivity, as diagnostic tests to identify causative organism are infrequently performed in clinical practice. We therefore included estimates for ACP. Estimates for ACP will have in contrast overestimated the burden caused by pneumococcus, as the majority of ACP is thought to be attributed to viral pathogens [57]. Information on causative pathogen or pneumococcal serotype is not available in the InGef database.

In addition, there are further limitations that relate to the structure of the InGef database. In the outpatient data, only yearly quarters in which diagnoses were made were available, not exact diagnosis dates which may have underestimated the number of PD episodes in the outpatient data. Antibiotic prescriptions during the quarters with the diagnoses were assumed to be related to the PD diagnosis to assign exact diagnosis dates thus potentially overestimating the number of antibiotic prescriptions being prescribed as a result of a PD episode.

The purpose of the present study was to estimate the direct medical costs associated with PD in Germany. Wider societal, vaccination or other indirect costs were therefore not assessed in the present study, but are nevertheless important when considering the broader economic burden of PD. Thus, the burden of PD reported in the present study is likely an underestimate.

## Conclusion

In Germany, between 2014 and 2019, HCRU and average cost per episode did not vary significantly for pneumonia and IPD, but for AOM, the annual cost per episode increased. The economic burden of pneumonia, IPD and AOM remains substantial in children aged < 16 years in Germany.

## Supplementary Information

**Additional file 1: Supplementary Table 1.** Definitions of pneumonia cases. **Supplementary Table 2.** Definitions of IPD cases. **Supplementary Table 3.** Definitions of AOM cases.

## Data Availability

The data that support the findings of this study are stored within the Institute for Applied Health Research Berlin GmbH (InGef, www.InGef.de). Restrictions apply to the availability of these data, and they are not publicly available. Access to patient-level data is not possible and all analyses must be conducted by InGef. Requests for bespoke analyses/ aggregate results are reviewed and approved by InGef.

## Abbreviations

ACP: All-cause pneumonia
AOM: Acute otitis media
CI: Confidence interval
HCRU: Healthcare resource utilization
InGef: Institute for Applied Health Research, Berlin
IPD: Invasive pneumococcal disease
PCV: Pneumococcal conjugate vaccine
PD: Pneumococcal disease
PP: Pneumococcal pneumonia
PY: Person-years
SD: Standard deviation
SHI: Statutory health insurance providers
S. pneumoniae: Streptococcus pneumoniae
STIKO: German standing committee on vaccination
