# Supplementary material for: Healthcare resource utilization and cost of pneumococcal disease in children in Germany, 2014–2019: a retrospective cohort study
Source: Pneumonia (Nathan). 2023 Mar 25;15:7. doi: 10.1186/s41479-023-00105-9 (PMC10039501; doi:10.1186/s41479-023-00105-9)
Supplement: Supplementary file 1 — Additional file 1: Supplementary Table 1. Definitions of pneumonia cases. Supplementary Table 2. Definitions of IPD cases. Supplementary Table 3. Definitions of AOM cases. [file 41479_2023_105_MOESM1_ESM.docx]

# Supplementary Information

**Supplementary Table 1. Definitions of pneumonia cases**

| Categories | Diagnosis | ICD 10 GM code |
| --- | --- | --- |
| PP | PP | J13 |
|  | Bacterial pneumonia, unspecified + PP | J15.9 + B95.3 |
|  | Bronchopneumonia, organism unspecified + PP | J18.0 + B95.3 |
|  | Lobar pneumonia unspecified + PP | J18.1 + B95.3 |
|  | Pneumonia organism unspecified + PP | J18.8 + B95.3 |
|  | Pneumonia organism unspecified + PP | J18.9 + B95.3 |
| All-cause pneumonia | Bacterial pneumonia, unspecified | J15.9 |
| *(PP+ All-cause pneumonia codes)* | Bronchopneumonia, organism unspecified | J18.0 |
|  | Lobar pneumonia unspecified | J18.1 |
|  | Pneumonia organism unspecified | J18.8 |
|  | Pneumonia organism unspecified | J18.9 |
|  | Influenza with pneumonia, seasonal influenza virus identified | J10.0 |
|  | Influenza with pneumonia, virus not identified | J11.0 |
|  | Viral pneumonia, not elsewhere classified | J12 |
|  | Pneumonia due to Haemophilus influenzae | J14 |
|  | Other bacterial pneumonia (Legionnaires' disease) | A48.1 |
|  | Other bacterial pneumonia | J15.0 |
|  | Other bacterial pneumonia | J15.1 |
|  | Other bacterial pneumonia | J15.2 |
|  | Other bacterial pneumonia | J15.3 |
|  | Other bacterial pneumonia | J15.4 |
|  | Other bacterial pneumonia | J15.5 |
|  | Other bacterial pneumonia | J15.6 |
|  | Other bacterial pneumonia | J15.7 |
|  | Other bacterial pneumonia | J15.8 |
|  | Pneumonia due to other infectious organisms, not elsewhere classified | J16 |
|  | Pneumonia in infectious diseases classified elsewhere | A22.1 |
|  | Whooping Cough | A37 |
|  | Pneumonia in infectious diseases classified elsewhere | B25.0 |
|  | Pneumonia in infectious diseases classified elsewhere | B44.0 |
|  | Pneumonia in diseases classified elsewhere | J17 |
|  | Pneumonia, organism unspecified | J18 |

**Supplementary Table 2. Definitions of IPD cases**

| **Category and ICD-10 GM** |
| --- |
| ***IPD Definitions*** |
| *Bacteremia*  A40.3, A40.9 + B95.3, A41.9 + B95.3, A49.9 + B95.3 |
| *Meningitis*  G00.1, G00.2 + B95.3, G00.9 + B95.3, G03.9 + B95.3 |
| *Bacteremic pneumonia*  A22.1 + A40.3, A22.1 + A40.9 + B95.3, A22.1 + A41.9 + B95.3, A22.1 + A49.9 + B95.3, A37 + A40.3, A37 + A40.9 + B95.3, A37 + A41.9 + B95.3, A37 + A49.9 + B95.3, A48.1 + A40.3, A48.1 + A40.9 + B95.3, A48.1 + A41.9 + B95.3, A48.1 + A49.9 + B95.3, B25.0 + A40.3, B25.0 + A40.9 + B95.3, B25.0 + A41.9 + B95.3, B25.0 + A49.9 + B95.3, B44.0 + A40.3, B44.0 + A40.9 + B95.3, B44.0 + A41.9 + B95.3, B44.0 + A49.9 + B95.3, J09 + A40.3, J09 + A40.9 + B95.3, J09 + A41.9 + B95.3, J09 + A49.9 + B95.3, J10.0 + A40.3, J10.0 + A40.9 + B95.3, J10.0 + A41.9 + B95.3, J10.0 + A49.9 + B95.3, J11.0 + A40.3, J11.0 + A40.9 + B95.3, J11.0 + A41.9 + B95.3, J11.0 + A49.9 + B95.3, J12 + A40.9 + B95.3, J12 + A41.9 + B95.3, J12 + A49.9 + B95.3, J12+ A40.3, J13 + A40.3, J13 + A40.9, J13 + A40.9 + B95.3, J13 + A41.9, J13 + A41.9 + B95.3, J13 + A49.9, J13 + A49.9 + B95.3, J14 + A40.3, J14 + A40.9 + B95.3, J14 + A41.9 + B95.3, J14 + A49.9 + B95.3, J15 + A40.3, J15 + A40.9 + B95.3, J15 + A41.9 + B95.3, J15 + A49.9 + B95.3, J16 + A40.3, J16 + A40.9 + B95.3, J16 + A41.9 + B95.3, J16 + A49.9 + B95.3, J17 + A40.3, J17 + A40.9 + B95.3, J17 + A41.9 + B95.3, J17 + A49.9 + B95.3, J18.0 + A40.3, J18.0 + A40.9 + B95.3, J18.0 + A41.9 + B95.3, J18.0 + A49.9 + B95.3, J18.1 + A40.3, J18.1 + A40.9 + B95.3, J18.1 + A41.9 + B95.3, J18.1 + A49.9 + B95.3, J18.8 + A40.3, J18.8 + A40.9 + B95.3, J18.8 + A41.9 + B95.3, J18.8 + A49.9 + B95.3, J18.9 + A40.3, J18.9 + A40.9 + B95.3, J18.9 + A41.9 + B95.3, J18.9 + A49.9 + B95.3, J85.1 + B95.3, J86.0 + B95.3, J86.9 + B95.3, A22.1 + A40.9 , A22.1 + A41.9, A22.1 + A49.9, A37 + A40.9, A37 + A41.9, A37 + A49.9, A48.1 + A40.9, A48.1 + A41.9, A48.1 + A49.9, B25.0 + A40.9 , B25.0 + A41.9, B25.0 + A49.9, B44.0 + A40.9, B44.0 + A41.9, B44.0 + A49.9, J09 + A40.9, J10.0 + A40.9, J10.0 + A41.9, J10.0 + A49.9, J11.0 + A40.9, J11.0 + A41.9, J11.0 + A49.9, J12 + A41.9, J12 + A49.9, J13 + A40.9, J14 + A40.9, J14 + A41.9, J14 + A49.9, J15 + A40.9, J15 + A41.9, J15 + A49.9, J16 + A40.9, J16 + A41.9, J16 + A49.9, J17 + A40.9, J17 + A41.9, J17 + A49.9, J18.0 + A40.9, J18.0 + A41.9, J18.0 + A49.9, J18.2 + A40.9, J18.2 + A41.9, J18.2 + A49.9, J18.8 + A40.9, J18.8 + A41.9, J18.8 + A49.9, J18.9 + A40.9, J18.9 + A41.9, J18.9 + A49.9 |
| *Other IPD*  I30.1 + B95.3, I33.0 + B95.3, I33.9 + B95.3, K65.0 + B95.3, K65.2 + B95.3, K65.8 + B95.3, K65.8 + B95.3, K65.9 + B95.3, M00.0 + B95.3, M00.1, M00.1 + B95.3, M00.2 + B95.3, M00.8 + B95.3 , M00.9 + B95.3, M86.1 + B95.3, M86.2 + B95.3, M86.9 + B95.3 |

**Supplementary Table 3. Definitions of AOM cases**

| **Categories** | **Diagnosis** | **ICD 10 GM codes** |
| --- | --- | --- |
| AOM | Suppurative and unspecified otitis media | H66 |
| AOM | Otitis media in diseases classified elsewhere | H67 |
